# Daily allergy burden and heart rate characteristics in adults with allergic rhinitis based on a wearable telemonitoring system

**DOI:** 10.1002/clt2.12242

**Published:** 2023-04-24

**Authors:** Joren Buekers, Michiel Stas, Raf Aerts, Nicolas Bruffaerts, Sebastien Dujardin, An Van Nieuwenhuyse, Jos Van Orshoven, Guillaume Chevance, Ben Somers, Jean‐Marie Aerts, Judith Garcia‐Aymerich

**Affiliations:** ^1^ ISGlobal Barcelona Spain; ^2^ Universitat Pompeu Fabra (UPF) Barcelona Spain; ^3^ CIBER Epidemiología y Salud Pública (CIBERESP) Barcelona Spain; ^4^ Measure, Model & Manage Bioresponses (M3‐BIORES) Department of Biosystems KU Leuven Leuven Belgium; ^5^ Division of Forest, Nature and Landscape Department of Earth and Environmental Sciences KU Leuven Leuven Belgium; ^6^ Risk and Health Impact Assessment Sciensano (Belgian Institute of Health) Brussels Belgium; ^7^ Division of Ecology, Evolution and Biodiversity Conservation KU Leuven Leuven Belgium; ^8^ Centre for Environmental Sciences University of Hasselt Hasselt Belgium; ^9^ KU Leuven Plant Institute KU Leuven Leuven Belgium; ^10^ Mycology and Aerobiology Sciensano (Belgian Institute of Health) Brussels Belgium; ^11^ Department of Geography University of Namur Namur Belgium; ^12^ Institute for Life, Earth and Environment (ILEE) University of Namur Namur Belgium; ^13^ Centre of Environment and Health Department of Public Health and Primary Care KU Leuven Leuven Belgium; ^14^ Department of Health Protection Laboratoire National de Santé (LNS) Dudelange Luxembourg; ^15^ KU Leuven Urban Studies Institute KU Leuven Leuven Belgium

**Keywords:** allergic rhinitis, heart rate, smartphone, symptoms, telemonitoring

## Abstract

**Background:**

Allergic rhinitis includes a certain degree of autonomic imbalance. However, no information is available on how daily changes in allergy burden affect autonomic imbalance. We aimed to estimate associations between daily allergy burden (allergy symptoms and mood) and daily heart rate characteristics (resting heart rate and sample entropy, both biomarkers of autonomic balance) of adults with allergic rhinitis, based on real‐world measurements with a wearable telemonitoring system.

**Methods:**

Adults with a tree pollen allergy used a smartphone application to self‐report daily allergy symptoms (score 0–44) and mood (score 0–4), and a Mio Alpha 2 wristwatch to collect heart rate characteristics during two pollen seasons of hazel, alder and birch in Belgium. Associations between daily allergy burden and heart rate characteristics were estimated using linear mixed effects distributed lag models with a random intercept for individuals and adjusted for potential confounders.

**Results:**

Analyses included 2497 participant‐days of 72 participants. A one‐point increase in allergy symptom score was associated with an increase in next‐day resting heart rate of 0.08 (95% CI: 0.02–0.15) beats per minute. A one‐point increase in mood score was associated with an increase in same‐day sample entropy of 0.80 (95% CI: 0.34–1.26) × 10^−2^. No associations were found between allergy symptoms and heart rate sample entropy, nor between mood and resting heart rate.

**Conclusion:**

Daily repeated measurements with a wearable telemonitoring system revealed that the daily allergy burden of adults with allergic rhinitis has systemic effects beyond merely the respiratory system.

## 1 INTRODUCTION

Allergic rhinitis is a major concern for public health. It is a chronic condition that involves inflammation of the nasal mucous membrane after exposure to specific allergens such as pollen, animal dander, or dust mites derived allergens.^1^ The prevalence of allergic rhinitis has increased over the last decades, with current prevalence estimates of up to 40% of the global population.^2^ Key symptoms include nasal itching and congestion, rhinorrhoea, sneezing, itching and redness of the eyes, and tearing.^3^ Besides the physical burden of experiencing allergy symptoms, allergic rhinitis also represents a psychological burden with detrimental effects on mood and mental health.^4^, ^5^, ^6^ The high prevalence, substantial economic costs, often life‐long morbidity and negative impact on daily functioning, sleep, mood, mental health, and quality of life all contribute to the high burden of allergic rhinitis.^7^, ^8^, ^9^

Allergic rhinitis involves effects beyond the respiratory system, including a certain degree of autonomic imbalance (i.e. imbalance between sympathetic and parasympathetic nervous system activity).^10^, ^11^ A well‐balanced autonomic system is the result of a complex, non‐linear interplay between the sympathetic and parasympathetic nervous system, which enables the cardiovascular system to adapt to constantly changing physical and psychological stressors.^12^ A well‐balanced autonomic system thus results in complex and non‐linear heart rate dynamics, which has traditionally been quantified through time‐ and/or frequency‐domain analyses of beat‐to‐beat intervals (i.e., conventional heart rate variability metrics).^13^ The non‐linear behavior of heart rate dynamics can additionally be captured by non‐linear metrics, such as sample entropy, applied to time series of beat‐to‐beat intervals (in milliseconds)^12^, ^13^ or heart rate (in beats per minute).^14^ Higher heart rate sample entropy values indicate a more irregular time series, and thus represents a better adaptive capacity of the cardiovascular system to physical and psychological stressors,^15^ enabled by a well‐balanced autonomic system. In contrast, elevated resting heart rates can be a sign of autonomic imbalance through excessive sympathetic activity and/or reduced parasympathetic activity.^16^, ^17^

Biomarkers of autonomic imbalance, such as elevated resting heart rate and altered heart rate variability, are established risk factors for hypertension, hyperglycaemia, cardiovascular disease, and mortality.^16^, ^18^, ^19^, ^20^ Resting heart rate and heart rate variability also respond to acute stressors, such as illness onset^21^ or psychological stressors,^22^, ^23^ in the general population. However, and despite the fact that adults with allergic rhinitis are constantly exposed to acute physical and psychological stressors, no information is available on how the constantly changing physical and psychological allergy burden in allergic rhinitis affects their heart rate and autonomic balance.

Telemonitoring systems, including smartphone applications and advancing wearable technology, provide a unique opportunity to address this knowledge gap. Allergic rhinitis has already been a crucial case study for the development and implementation of smartphone applications to monitor real‐world disease control, including daily measurements of allergy symptoms and medication intake.^24^, ^25^, ^26^, ^27^ Wearable devices could additionally be used to measure heart rate characteristics that are indicative of autonomic (im)balance, such as resting heart rate^28^, ^29^ and heart rate sample entropy.^14^ Hence, daily repeated measurements of these heart rate characteristics can allow us to examine day‐to‐day changes in autonomic balance.

Therefore, this study aimed to examine associations between daily allergy burden (allergy symptoms and mood) and daily heart rate characteristics (resting heart rate and sample entropy) of adults with allergic rhinitis over the course of a pollen season, based on measurements with a wearable telemonitoring system. Allergy burden was assessed based on allergy symptoms and mood to represent both the physical and psychological burden of allergic rhinitis, respectively. We hypothesised that high allergy burden (i.e. high allergy symptoms and low mood) of adults with allergic rhinitis is associated with high autonomic imbalance (i.e. high resting heart rate and low sample entropy) on the same and next day(s).

## 2 MATERIAL AND METHODS

### 2.1 Study design and participants

RespirIT was an intensive longitudinal panel study with daily telemonitoring of allergy symptoms, mood, medication intake, GPS location, and heart rate during the pollen seasons of hazel, alder and birch (January–May) of 2017 and/or 2018 in Belgium. Adults were recruited from the general population in Belgium via advertisements on the airallergy.be website, on social media, in newspapers distributed for free on the national railway network, and via direct mailing. Inclusion criteria for participation were (a) being over 20 years old, (b) living in Belgium, (c) being sensitised to pollen of common hazel (*Corylus avellana*), alder (*Alnus* spp.) and/or birch (*Betula* spp.), and (iv) agreeing to use a wearable telemonitoring system. The allergic sensitisation was self‐reported, similar to the strategy adopted by the Mobile Airways Sentinel networK (MASK, the Phase 3 ARIA initiative).^25^ This strategy allowed the recruitment of a heterogeneous sample of adults with allergic rhinitis that is more representative of a routine care context than adults recruited in a clinical setting, as most adults with allergic rhinitis self‐medicate when they experience symptoms.^30^, ^31^ There were no additional exclusion criteria for participation, in line with previous MASK studies,^24^, ^32^ and adults following immunotherapy were not excluded.

The telemonitoring system consisted of: (a) a Samsung Galaxy J1 smartphone with the RespirIT application installed to self‐report daily allergy symptoms, mood, and medication intake, and (b) a Mio Alpha 2 wristwatch that measured daytime heart rate at a frequency of 1 Hz. Every participant received a personal oral explanation on how to use the wearables and how to operate the RespirIT application. Participants were asked to wear the wristwatch every day and during all activities (unless the watch interfered with work safety, hygiene protocols, or during stays abroad) from the moment they woke up until they went to sleep. Heart rate data were transmitted to the smartphone via a Bluetooth connection. All data were synchronised with a database in the cloud when the smartphone was connected to Wi‐Fi. A reminder to start the heart rate measurements was sent at the beginning of every day, and a reminder to report daily allergy symptoms, mood, and medication intake was sent at the end of every day. The research team sporadically checked whether data was being uploaded to the cloud database, and participants were contacted by email if this was not the case. No further mechanisms were implemented to increase adherence to the study protocol.

Participants with less than 10 valid days (defined as days with at least 6 h of heart rate data and information available on allergy symptoms, mood, and medication intake) were considered non‐compliant and were therefore excluded. The study was approved by the Ethical Commission of the KU Leuven University Hospital (Belgian registration number B322201629692). Participants provided written informed consent.

### 2.2 Daily allergy symptoms, mood, and medication intake

At the end of every day, participants logged their daily allergy symptoms, mood, and medication intake in the smartphone application. First, participants answered the question ‘What symptoms have been bothering you today and to what degree?’ by using sliders with discrete values from 0 (never) to 4 (always) for each of the following 11 symptoms that are related to seasonal allergic rhinitis: wheezing, dyspnoea, coughing, sneezing, runny or stuffy nose, itching, fatigue, headache, bad sleep, difficulty concentrating, and irritation of the eyes. Daily allergy symptoms were scored between 0 and 44 by summing the individual values of these 11 symptoms.^33^ Second, daily mood was interpreted as the general feeling of the participants during the considered day and was not specifically tailored to allergic rhinitis. Participants answered the question ‘How did you feel today?’ on a five‐point rating scale, which was translated into a daily mood score between 0 (worst) and 4 (best).^6^ Third, participants listed medications they took that specific day. These medications were divided into the following medication classes: antihistamines, corticosteroids, and/or decongestants.

### 2.3 Daily heart rate characteristics

Heart rate time series were pre‐processed in three sequential steps (Python code on https://osf.io/ngyzv/) with the aim to reduce the noise in the time series and to better capture the intrinsic speed of the heart rate dynamics. First, missing data were linearly interpolated when the gap of missing data was shorter than 20 s. Second, heart rate data were smoothed using locally weighted scatterplot smoothing with a bandwidth of 60 s.^14^ Third, the data were down‐sampled to a sampling time of 10 s (0.1 Hz) by taking the centered mean of every 10 s of heart rate data.

Two heart rate characteristics were calculated for every day based on daytime heart rate data: resting heart rate and sample entropy (Python code on https://osf.io/ngyzv/). Resting heart rate was calculated for every day as the minimal value of a 5 min rolling mean.^34^ An elevated resting heart rate can represent autonomic imbalance and is a risk factor for different chronic diseases and mortality.^18^, ^19^ Sample entropy was calculated as described by Richman and Moorman,^35^ using an embedding dimension (m) of 2 and an individualised tolerance (r) of 0.15 times the standard deviation of all heart rate data of the considered participant.^14^ Only the first 6 h of heart rate data per day (i.e. 6 h including 60 min per hour, including 6 data points per minute = 2160 data points) were used to calculate sample entropy to exclude unwanted variability due to inequal time series lengths.^36^ Higher sample entropy values indicate a more irregular time series. Consequently, a high heart rate sample entropy represents a better adaptive capacity to chronic and/or acute stressors.^15^

Within‐participant outliers of daily resting heart rate or sample entropy values were removed. These outliers were identified as values outside 1.5 times the interquartile range below/above the first/third quartile of all available resting heart rate or sample entropy values of the considered participant.^37^

### 2.4 Other relevant characteristics

Participants self‐reported information on age, sex, body mass index, current smoking status (smoker vs. non‐smoker), frequency of physical activity (less vs. at least 2 times per week 20 min of activity), short‐term emotional distress (12‐item General Health Questionnaire), chronic respiratory problems (asthma and/or chronic respiratory disease: yes vs. no), additional allergies (grass and/or dust mites allergy: yes vs. no), and general medication use (antihistamines and/or corticosteroids: yes vs. no). Green space cover in a 1 km radius around the residence was determined as the cumulative area of garden, grassland, and forest.^6^ A previous RespirIT study calculated personalised daily exposure to nitrogen dioxide (NO_2_), ozone (O_3_) and particulate matter with diameter less than 10 μm (PM_10_) for 2–24 days (median of 6 days) per participant.^33^ An estimate of average exposure to NO_2_, O_3_ and PM_10_ was calculated per participant as the mean of these 2–24 days. The start and end dates of the pollen seasons of hazel, alder and birch were determined with the AeRobiology R software package,^38^ based on the method described by Pfaar et al.^39^

Time spent in moderate‐to‐vigorous physical activity was estimated for every day as the amount of time spent with a heart rate reserve above 40% (= resting heart rate + (maximal heart rate – resting heart rate) × 40%),^40^ to control for the potential influence of physical activity on heart rate characteristics.

### 2.5 Statistical analyses

The minimal sample size was estimated with G*Power 3.1.9.7. Using a two‐sided *α* = 0.05, a power of 90% and assuming a small effect size (*f* ^2^ = 0.01), a sample size of 1422 participant‐days would be sufficient to detect statistically significant associations between daily allergy burden and daily heart rate characteristics.

Participant characteristics and daily heart rate characteristics, allergy burden, physical activity, and medication intake are presented as n (%) for categorical variables and as mean (standard deviation) or median (interquartile range) for continuous variables with normal or non‐normal distributions, respectively.

Associations between daily allergy burden (at lags of 0, 1, and 2 days; exposure variables) and daily heart rate characteristics (outcome variables) were estimated using linear mixed effects models with random intercepts for participants. Allergy burden at lags of 0, 1, and 2 days represent the allergy burden on respectively the same‐day, previous‐day and 2 days before, relative to the day of the considered heart rate characteristics. Models were adjusted for age, sex, additional allergy to grass and/or dust mites, time spent in moderate‐to‐vigorous physical activity, exposure to NO_2_, exposure to PM_10_, medication intake, month, weekend days, and pollen seasons of birch, alder and hazel. A first‐order autocorrelation structure for the residuals was added to account for autocorrelation in the model residuals. Exposure variables were included one at a time (single‐exposure models) or both together (multi‐exposure models) in distributed lag models (i.e. including lags of 0, 1, and 2 days together). When a statistically significant association was found between allergy symptoms and a heart rate characteristic, a post‐hoc analysis was performed to determine the associations between each of the 11 different symptoms and the considered heart rate characteristic. Single‐ and multi‐exposure models including only one lag of the exposure variables at a time (i.e. single lag model) were used in a sensitivity analysis.

Data preparation, heart rate pre‐processing, and calculations of daily heart rate characteristics, allergy symptom scores and mood scores were performed in JupyterLab using the Python 3.5 programming language. Statistical analyses were performed in JupyterLab using the R 4.1.2 programming language (nlme package).

## 3 RESULTS

**FIGURE 1:**
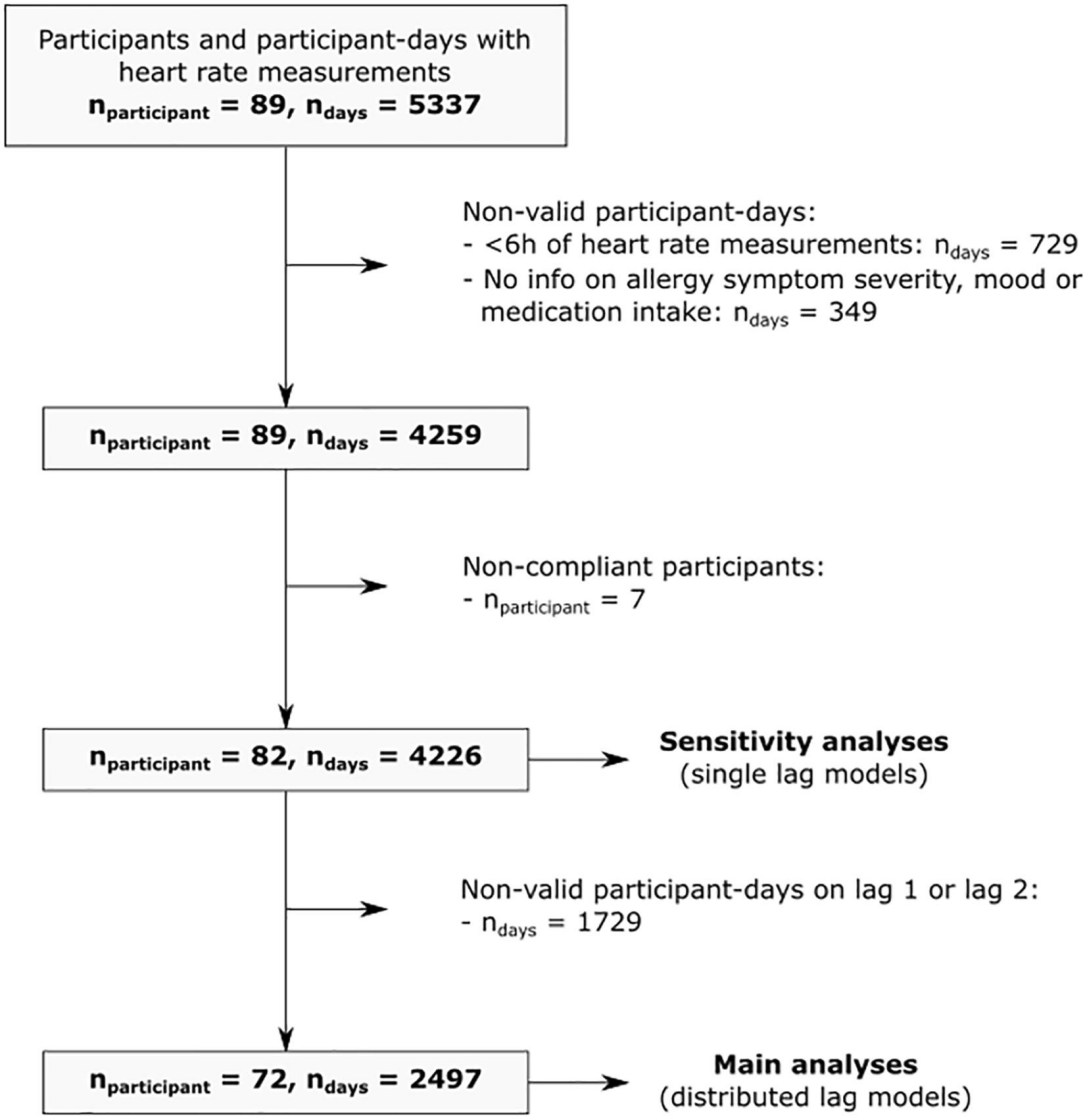
Flow diagram of included participants and participant‐days in the main and sensitivity analyses. Valid participant days were defined as days with at least 6 h of heart rate data and information available on allergy symptoms, mood, and medication intake. Participants with less than 10 valid days were considered non‐compliant.

Data on a total of 72 participants and 2497 participant‐days were included in the main analyses (Figure 1). Participants were mostly female (65%) with a mean ± SD age of 40 ± 11 years (Table 1). Most participants were also allergic to grass pollen and/or dust mites (74%), and had no other chronic respiratory problems besides allergic rhinitis (82%). Participants used antihistamines (*n* = 45), corticosteroids (*n* = 3), or both antihistamines and corticosteroids (*n* = 20). Very few participants did not use standard allergy medication (*n* = 4). On average 35 participant‐days were included per participant (minimum: 6, maximum: 116). Table 2 summarises the heart rate characteristics, allergy symptom score, mood score, time spent in moderate‐to‐vigorous physical activity, and medication intake for all 2497 included participant‐days. Women had a higher resting heart rate (65 ± 6 beats per minute vs. 59 ± 7 beats per minute; *p* = 0.001) and sample entropy (0.45 ± 8 vs. 0.40 ± 10; *p* = 0.02) than men. All values were similar in the sensitivity analysis dataset that included 4226 participant‐days from 82 participants (Appendix).

**TABLE 1:** Participant characteristics, presented as mean (standard deviation) or *n* (%).

	Participants (*n* = 72)
Sex, female – male, *n* (%)	47 (65)–25 (35)
Age (years)	
Continuous, mean (SD)	40 (11)
<30, *n* (%)	14 (19)
30–39, *n* (%)	22 (31)
40–49, *n* (%)	19 (26)
≥50, *n* (%)	17 (24)
Body Mass index (kg·m^−2^), mean (SD)	24.4 (4.6)
Current smoker, *n* (%)	2 (3)
Physical activity, < 2 × 20’/week, *n* (%)	12 (19)
Short‐term emotional distress (GHQ‐12 score)	
Continuous, median (IQR)	1 (3)
<2, *n* (%)	36 (58)
≥2, *n* (%)	26 (42)
Chronic respiratory problems	
Asthma, *n* (%)	11 (15)
Chronic respiratory disease, *n* (%)	0 (0)
Both, *n* (%)	2 (3)
Neither, *n* (%)	59 (82)
Additional allergies	
Grass, *n* (%)	24 (33)
Dust mites, *n* (%)	6 (8)
Both, *n* (%)	23 (32)
Neither, *n* (%)	19 (26)
Medication	
Antihistamines, *n* (%)	45 (62)
Corticosteroids, *n* (%)	3 (4)
Both, *n* (%)	20 (28)
Neither, *n* (%)	4 (6)
Residential green space cover within 1 km (ha), mean (SD)	151 (60)
Exposure to air pollution	
Nitrogen dioxide (μg/m^3^), mean (SD)	23 (7)
Ozone (μg/m^3^), mean (SD)	50 (9)
Particulate matter with diameter less than 10 μm (μg/m^3^), mean (SD)	22 (5)

Abbreviation: GHQ‐12, 12‐item General Health Questionnaire.

**TABLE 2:** Heart rate characteristics, allergy burden, physical activity and medication intake for 2497 included participant‐days.

	Participant‐days (*n* = 2497)
Heart rate characteristics	
Resting heart rate (beats per minute), mean (SD)	62 (8)
Sample entropy, mean (SD)	0.42 (0.11)
Allergy burden	
Allergy symptoms (score 0–44), median ([IQR]; min–max)	1 ([0–5] 5; 0–37)
Mood (score 0–4), median ([IQR]; min–max)	3 (3–3; 0–4)
Physical activity	
Moderate‐to‐vigorous physical activity (minutes), median [IQR]	34 [14–64]
Medication taken	
Antihistamine, *n* (%)	1070 (43)
Corticosteroid, *n* (%)	519 (21)
Decongestant, *n* (%)	18 (1)

**FIGURE 2:**
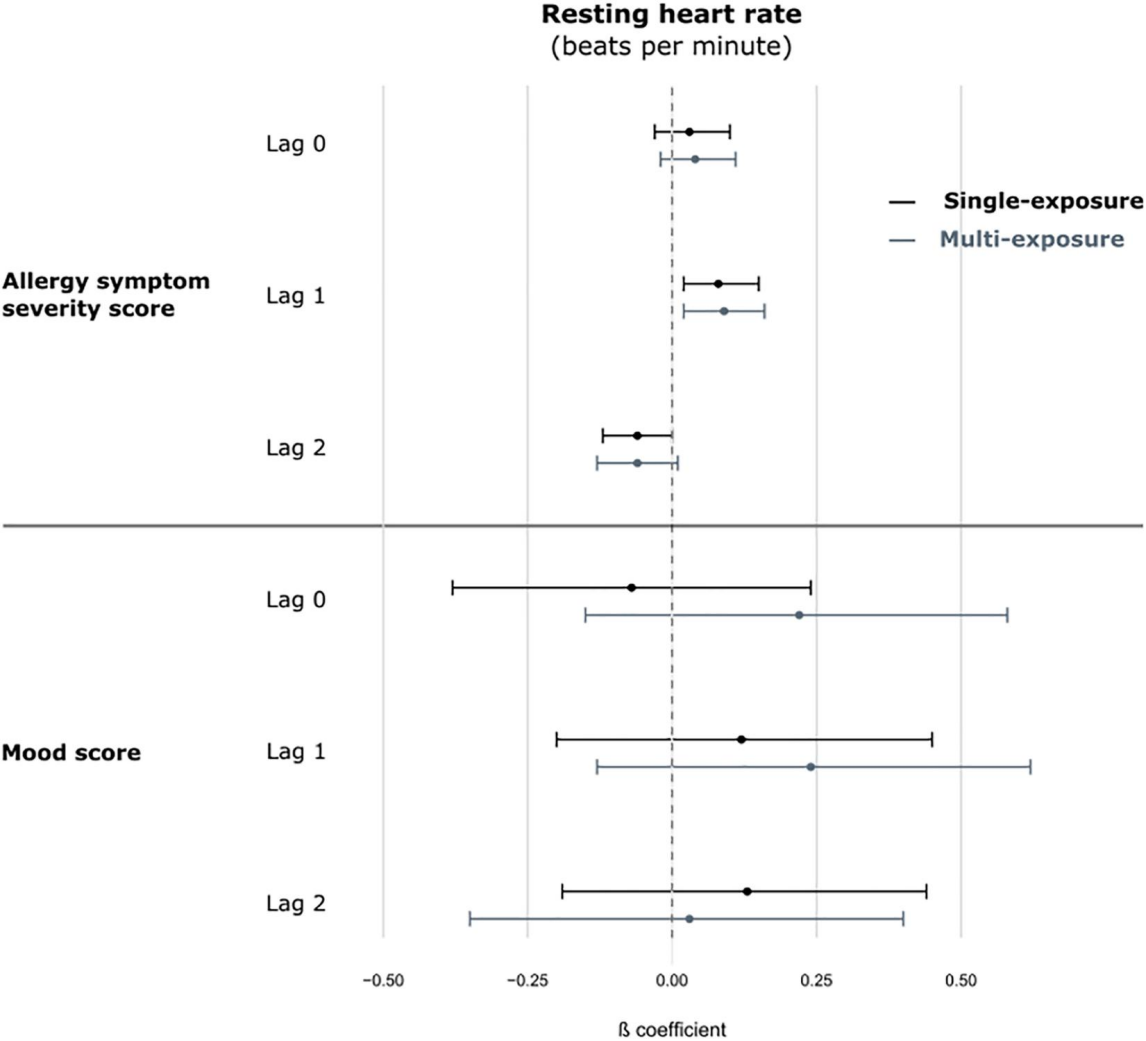
Associations between allergy symptoms and mood, and resting heart rate expressed as *β* coefficient (95% CI) of a linear mixed effects model with random intercepts for participants and including exposure variables at lags of 0, 1, and 2 days together (distributed lag model). Higher allergy symptom score indicates more severe symptoms, higher mood score indicates better mood. Models were adjusted for age, sex, additional allergy to grass and/or dust mites, time spent in moderate‐to‐vigorous physical activity, exposure to nitrogen dioxide, exposure to particulate matter with diameter less than 10 μm, medication intake, month, weekend days and pollen seasons of birch, alder and hazel. Also a first‐order autocorrelation structure for the residuals was added. Multi‐exposure models were additionally adjusted for the other exposure variable at lags of 0, 1 and 2 days.

A one‐point increase in allergy symptom score was associated with an increase in next‐day resting heart rate of 0.08 (95% CI: 0.02–0.15) and 0.09 (95% CI: 0.02–0.16) beats per minute in the single‐ and multi‐exposure distributed lag models, respectively (Figure 2 and Table A1 in Appendix). Post‐hoc analyses indicated that mainly previous‐day wheezing [0.78 (95% CI: 0.22–1.34) and 0.79 (95% CI: 0.23–1.35) in the single‐ and multi‐exposure distributed lag models, respectively], sneezing [0.34 (95% CI: 0.04–0.64) and 0.36 (95% CI: 0.06–0.67)] and irritation of the eyes [0.29 (95% CI: 0.01–0.57) and 0.31 (95% CI: 0.03, 0.59)] contributed to this association. Mood was not associated with resting heart rate at any lag. Sensitivity analyses based on 4226 participant‐days from 82 participants in single lag models provided comparable results (Table A2 in Appendix).

**FIGURE 3:**
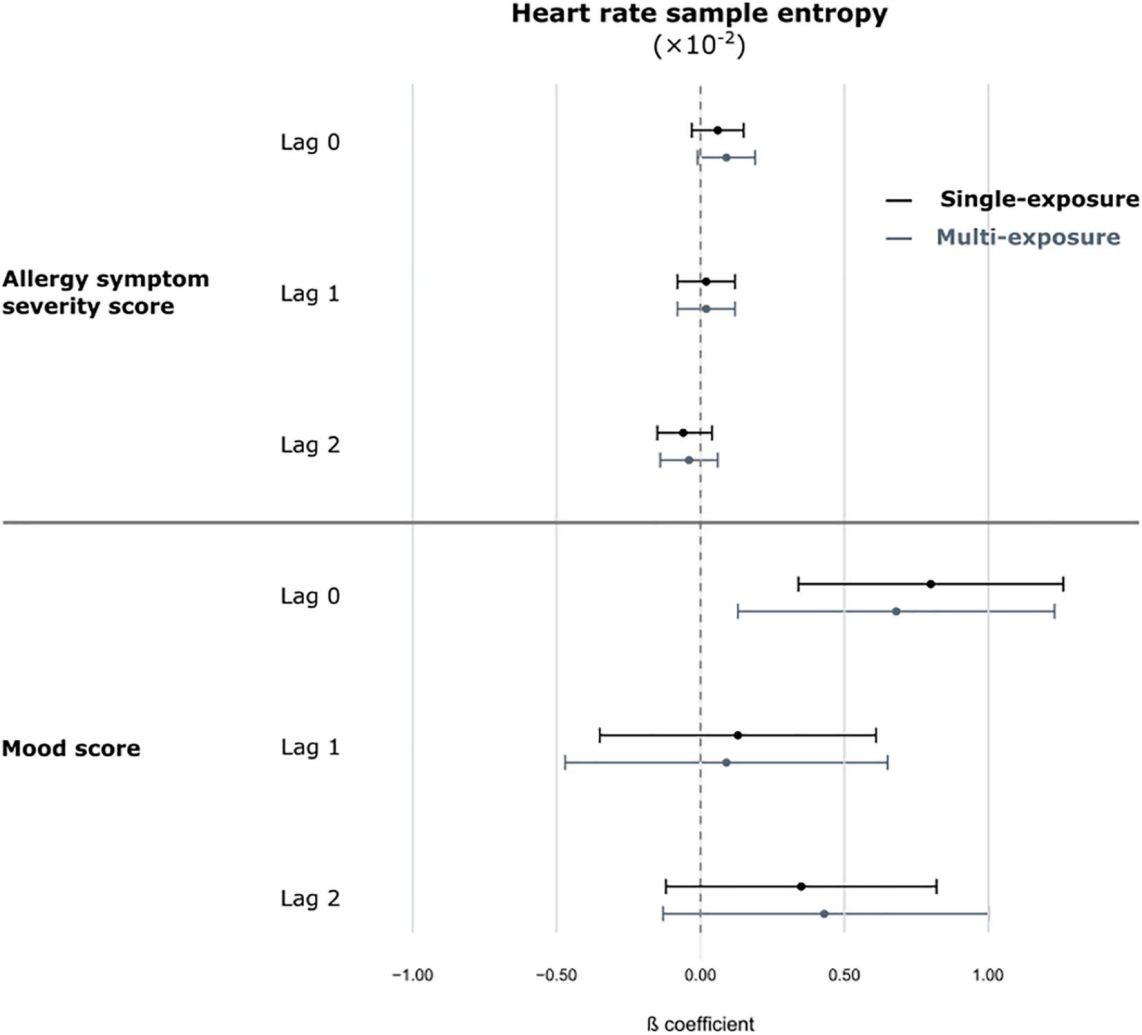
Associations between allergy symptoms and mood, and heart rate sample entropy expressed as β coefficient (95% CI) of a linear mixed effects model with random intercepts for participants and including exposure variables at lags of 0, 1, and 2 days together (distributed lag model). Higher allergy symptom score indicates more severe symptoms, higher mood score indicates better mood. Models were adjusted for age, sex, additional allergy to grass and/or dust mites, time spent in moderate‐to‐vigorous physical activity, exposure to nitrogen dioxide, exposure to exposure to particulate matter with diameter less than 10 μm, medication intake, month, weekend days and pollen seasons of birch, alder and hazel. Also a first‐order autocorrelation structure for the residuals was added. Multi‐exposure models were additionally adjusted for the other exposure variable at lags of 0, 1 and 2 days.

A one‐point increase in mood score was associated with an increase in same‐day sample entropy of 0.80 (95% CI: 0.34–1.26) × 10^−2^ and 0.68 (95% CI: 0.13–1.23) × 10^−2^ in the single‐ and multi‐exposure distributed lag models, respectively (Figure 3 and Table A3 in Appendix). Allergy symptoms were generally not associated with heart rate sample entropy. Sensitivity analyses using single lag models provided comparable results (Table A4 in Appendix).

## 4 DISCUSSION

This longitudinal study was the first to reveal an association between daily allergy burden and autonomic (im)balance in adults with allergic rhinitis. Daily repeated measurements with a wearable telemonitoring system indicated that (1) an increase in allergy symptom score was associated with an increase in next‐day resting heart rate, and (2) an increase in mood score was associated with an increase in same‐day heart rate sample entropy. No clear associations were found between allergy symptoms and heart rate sample entropy, nor between mood and resting heart rate.

The observed positive association between allergy symptoms and next‐day resting heart rate suggests that an acute allergy burden contributes to the altered autonomic balance in adults with allergic rhinitis. This supports our hypothesis that a high allergy burden can increase resting heart rate, in accordance to previous studies that observed increases in resting heart rate in the general population after exposure to negative stressors such as air pollution,^41^ acute pain,^42^ alcohol intake,^21^ or acute illness onset.^21^ Although the effect size of the total allergy symptom score was small, a one‐point increase in wheezing increased next‐day resting heart rate to a similar degree as the resting heart rate increase during a 5^th^–95th percentile change in air pollutant concentration,^41^ the resting heart rate increase the day after a high‐intensity training,^21^ or as the difference in resting heart rate between different stages of the menstrual cycle.^21^ The results furthermore imply that there is a lingering effect of allergy symptoms on next‐day autonomic balance. The trend toward a positive association between allergy symptoms and same‐day resting heart rate in the main and sensitivity analyses suggests that the effect of allergy symptoms on autonomic balance might already start on the same day. The trend toward a negative association between allergy symptoms and resting heart rate at lag 2 was not observed in the sensitivity analyses and might be due to chance.

The positive association between mood and same‐day sample entropy highlights the link between mental and physiological health. Previous studies identified lower time‐domain and non‐linear heart rate variability metrics, conceptually close to heart rate sample entropy, in patients with major depressive disorder compared to healthy peers.^43^ In bipolar patients, a non‐linear heart rate variability metric comparable to heart rate sample entropy increased when transitioning from a pathological to a healthy mood state.^44^ Our study found an association between more subtle, day‐to‐day mood changes and alterations in heart rate sample entropy (representative of autonomic balance), which can possibly only be detected by means of daily repeated measurements with a wearable telemonitoring system over a prolonged period of time. Of note, the association observed in our study might be the result of a bi‐directional relationship between heart rate sample entropy and mood.^45^

In contrast to our hypothesis, allergy symptoms were found not to be associated with heart rate sample entropy at any lag. Methodological differences (e.g. real‐life setting vs. provocation tests, heart rate sample entropy vs. conventional heart rate variability metrics) might explain the discrepancy between our results and previous studies that identified alterations in heart rate variability metrics after inducing allergy symptoms in a provocation test.^46^, ^47^

There are some research implications of our study. The developed methodology (Python code available on https://osf.io/ngyzv/) can allow future studies to examine changes in autonomic imbalance at a high resolution (i.e. day‐to‐day changes) based on heart rate measurements with affordable, user‐friendly wearable technology. This can reveal how different types of stressors can affect autonomic imbalance, and whether heart rate characteristics can be predictive of adverse events (e.g. allergy symptoms onset). A similar association between mood and heart rate sample entropy as found in our study could furthermore be expected in the general population, as our assessment of mood was not tailored to allergic rhinitis. From a clinical perspective, the results point out that allergic rhinitis and the daily allergy burden can have systemic effects beyond merely the respiratory system.

There were some limitations of the study. Firstly, allergy symptom and mood were self‐reported through a questionnaire that has not been formally validated, which may have added a certain level of unwanted variability in the data. Secondly, the recruitment strategy based on advertisements may have been a source of selection bias, as technophiles and/or active adults might be more inclined to participate in a study that makes use of wearable devices. This might have contributed to the relatively high adherence to the study protocol, in combination with the fact that participants were motivated to participate due to concerns about their allergy symptoms and/or interests in the outcomes of the study. In addition, adherence to the app might be higher on days when participants experience allergy symptoms.^32^ Nevertheless, the low allergy symptom scores for most days (median [IQR] of 1 [0–5]) indicates that adherence to the app was also high on days with little to no allergy symptoms. Thirdly, the low day‐to‐day variability in daily allergy burden might have reduced the statistical power of our analyses. Hence, the observed associations between daily allergy burden and heart rate characteristics might be an underestimation of the true associations. Fourthly, we could not test the role of decongestant intake on resting heart rate,^48^ due to the low proportion of participant‐days on which decongestants were taken (1% of days). Fifthly, sample entropy values depend on the chosen parameter values (m and r), and the sampling time and length of the heart rate time series. Hence, sample entropy is considered a relative metric.^36^ This complicates the interpretation of effect sizes, establishing minimal important differences and the comparison with values in other populations. Lastly, potential effect modification related to days with versus without intake of antihistamine, corticosteroid or decongestant could not be examined because this analysis would mainly stratify between participants (participants who regularly take antihistamines, corticosteroids or decongestant vs. those who do not) instead of stratifying between days with versus days without antihistamine, corticosteroid or decongestant intake within the same participant.

Important strengths of the study include the large dataset of 2497 participant‐days and the longitudinal design with on average 35 repeated measurements per participant. A cross‐sectional design might not have been able to detect the associations that were revealed by the daily repeated measurements in our study. Another strength is the novelty of the approach, combining convenient wearable technology with relatively simple algorithms that can translate continuous heart rate measurements into clinically relevant metrics. This approach does not require inconvenient electrocardiographic measurements of the time intervals between consecutive heart beats (in milliseconds) that would be needed to calculate conventional heart rate variability metrics,^12^ and can therefore easily be adopted in future telemonitoring studies that perform continuous heart rate measurements (in beats per minute) with user‐friendly wearable devices. The approach is furthermore representative of daily life reality in adults with allergic rhinitis, as opposed to conventional, laboratory‐based provocation tests.

This was the first study that used a wearable telemonitoring system to show that daily allergy symptoms and mood are associated with daily heart rate characteristics in adults with allergic rhinitis. A higher allergy symptom score was associated with an increased resting heart rate on the next day, while a higher mood score was related to a more irregular, healthier heart rate on the same day. These results emphasise the role of daily allergy symptoms and mood in the autonomic (im)balance of adults with allergic rhinitis. The presented approach can furthermore easily be adopted in future telemonitoring studies that aim to examine changes in autonomic imbalance at a high resolution.

## AUTHOR CONTRIBUTIONS

**Joren Buekers**: Formal analysis (Equal); Visualization (Lead); Writing – original draft (Lead); Writing – review & editing (Lead). **Michiel Stas**: Data curation (Equal); Project administration (Equal); Writing – review & editing (Equal). **Raf Aerts**: Data curation (Equal); Writing – review & editing (Equal). **Nicolas Bruffaerts**: Conceptualization (Equal); Data curation (Equal); Funding acquisition (Equal); Project administration (Equal); Writing – review & editing (Equal). **Sebastien Dujardin**: Writing – review & editing (Equal). **An Van Nieuwenhuyse**: Conceptualization (Equal); Data curation (Equal); Funding acquisition (Equal); Project administration (Equal); Writing – review & editing (Equal). **Jos Van Orshoven**: Conceptualization (Equal); Data curation (Equal); Funding acquisition (Equal); Project administration (Equal); Writing – review & editing (Equal). **Guillaume Chevance**: Writing – review & editing (Equal). **Ben Somers**: Conceptualization (Equal); Data curation (Equal), Funding acquisition (Equal), Project administration (Equal); Writing – review & editing (Equal). **Jean‐Marie Aerts**: Conceptualization (Equal); Data curation (Equal); Funding acquisition (Equal); Project administration (Equal); Writing – review & editing (Equal). **Judith Garcia‐Aymerich**: Supervision (Lead); Writing – original draft (Equal), Writing – review & editing (Equal).

## CONFLICT OF INTEREST STATEMENT

The authors declear no conflicts of interest.

## Supporting information

Supporting Information S1

## References

[1] Bousquet J , Anto JM , Bachert C , et al. Allergic rhinitis. Nat Rev Dis Prim. 2020;6(1):95. 10.1038/s41572-020-00227-0 33273461

[2] Katelaris CH , Lee BW , Potter PC , et al. Prevalence and diversity of allergic rhinitis in regions of the world beyond Europe and North America. Clin Exp Allergy. 2012;42(2):186‐207. 10.1111/j.1365-2222.2011.03891.x 22092947

[3] Brożek JL , Bousquet J , Agache I , et al. Allergic rhinitis and its impact on asthma (ARIA) guidelines—2016 revision. J Allergy Clin Immunol. 2017;140(4):950‐958. 10.1016/j.jaci.2017.03.050 28602936

[4] Marshall PS , O’Hara C , Steinberg P . Effects of seasonal allergic rhinitis on fatigue levels and mood. Psychosom Med. 2002;64(4):684‐691. 10.1097/01.psy.0000021944.35402.44 12140359

[5] Trikojat K , Luksch H , Rösen‐Wolff A , Plessow F , Schmitt J , Buske‐Kirschbaum A . “Allergic mood” – depressive and anxiety symptoms in patients with seasonal allergic rhinitis (SAR) and their association to inflammatory, endocrine, and allergic markers. Brain Behav Immun. 2017; 65: 202‐209. 10.1016/j.bbi.2017.05.005 28495610

[6] Stas M , Aerts R , Hendrickx M , et al. Residential green space types, allergy symptoms and mental health in a cohort of tree pollen allergy patients. Landsc Urban Plan. 2021:210: 104070. 10.1016/j.landurbplan.2021.104070

[7] Chen MH , Su TP , Chen YS , et al. Allergic rhinitis in adolescence increases the risk of depression in later life: a nationwide population‐based prospective cohort study. J Affect Disord. 2013; 145:49‐53. 10.1016/j.jad.2012.07.011 22889525

[8] Blaiss MS , Hammerby E , Robinson S , Kennedy‐Martin T , Buchs S . The burden of allergic rhinitis and allergic rhinoconjunctivitis on adolescents: a literature review. Ann Allergy Asthma Immunol [Internet]. 2018; 121:43‐52.e3. 10.1016/j.anai.2018.03.028 29626629

[9] Zuberbier T , Lötvall J , Simoens S , Subramanian SV , Church MK . Economic burden of inadequate management of allergic diseases in the European Union: a GA2LEN review. Allergy Eur J Allergy Clin Immunol. 2014;69(10):1275‐1279. 10.1111/all.12470 24965386

[10] Ishman SL , Martin TJ , Hambrook DW , Smith TL , Jaradeh SS , Loehrl TA . Autonomic nervous system evaluation in allergic rhinitis. Otolaryngol Head Neck Surg. 2007;136(1):51‐56. 10.1016/j.otohns.2006.08.014 17210333

[11] Taşcilar E , Yokusoglu M , Dundaroz R , et al. Cardiac autonomic imbalance in children with allergic rhinitis. Tohoku J Exp Med. 2009;219(3):187‐191. 10.1620/tjem.219.187 19851046

[12] Shaffer F , Ginsberg JP . An overview of heart rate variability metrics and norms. Front Public Health. 2017;5:1‐17. 10.3389/fpubh.2017.00258 29034226 PMC5624990

[13] Weippert M , Behrens M , Rieger A , Behrens K . Sample entropy and traditional measures of heart rate dynamics reveal different modes of cardiovascular control during low intensity exercise. Entropy. 2014;16(11):5698‐5711. 10.3390/e16115698

[14] Rector JL , Gijzel SMW , van de Leemput IA , van Meulen FB , Olde Rikkert MGM , Melis RJF . Dynamical indicators of resilience from physiological time series in geriatric inpatients: lessons learned. Exp Gerontol. 2021:149. 10.1016/j.exger.2021.111341 33838217

[15] Lipsitz LA , Goldberger AL . Loss of “complexity” and aging. JAMA. 1992;267(13):1806. 10.1001/jama.1992.03480130122036 1482430

[16] Wulsin LR , Horn PS , Perry JL , Massaro JM , D’Agostino RB . Autonomic imbalance as a predictor of metabolic risks, cardiovascular disease, diabetes, and mortality. J Clin Endocrinol Metab. 2015;100(6):2443‐2448. 10.1210/jc.2015-1748 26047073

[17] Thayer JF , Yamamoto SS , Brosschot JF . The relationship of autonomic imbalance, heart rate variability and cardiovascular disease risk factors. Int J Cardiol [Internet]. 2010;141:122‐131. 10.1016/j.ijcard.2009.09.543 19910061

[18] Seviiri M , Lynch BM , Hodge AM , et al. Resting heart rate, temporal changes in resting heart rate, and overall and cause‐specific mortality. Heart. 2018;104(13):1076‐1085. 10.1136/heartjnl-2017-312251 29269380

[19] Fox K , Borer JS , Camm AJ , et al. Resting heart rate in cardiovascular disease. J Am Coll Cardiol. 2007;50(9):823‐830. 10.1016/j.jacc.2007.04.079 17719466

[20] Tsuji H , Venditti FJ , Manders ES , et al. Reduced heart rate variability and mortality risk in an elderly cohort: the Framingham heart study. Circulation. 1994;90(2):878‐883. 10.1161/01.cir.90.2.878 8044959

[21] Altini M , Plews D . What is behind changes in resting heart rate and heart rate variability? A large‐scale analysis of longitudinal measurements acquired in free‐living. Sensors. 2021;21(23):21. 10.3390/s21237932 PMC8659706 34883936

[22] Schubert C , Lambertz M , Nelesen RA , Bardwell W , Choi JB , Dimsdale JE . Effects of stress on heart rate complexity‐A comparison between short‐term and chronic stress. Biol Psychol. 2009;80(3):325‐332. 10.1016/j.biopsycho.2008.11.005 19100813 PMC2653595

[23] Pereira T , Almeida PR , Cunha JPS , Aguiar A . Heart rate variability metrics for fine‐grained stress level assessment. Comput Methods Programs Biomed. 2017;148:71‐80. 10.1016/j.cmpb.2017.06.018 28774440

[24] Bousquet J , Devillier P , Arnavielhe S , et al. Treatment of allergic rhinitis using mobile technology with real‐world data: the MASK observational pilot study. Allergy Eur J Allergy Clin Immunol. 2018;73(9):1763‐1774. 10.1111/all.13406 29336067

[25] Bousquet J , Arnavielhe S , Bedbrook A , et al. Mask 2017: ARIA digitally‐enabled, integrated, person‐centred care for rhinitis and asthma multimorbidity using real‐world‐evidence. Clin Transl Allergy. 2018;8:1‐21. 10.1186/s13601-018-0227-6 30386555 PMC6201545

[26] Alvarez‐Perea A , Dimov V , Popescu FD , Zubeldia JM . The applications of eHealth technologies in the management of asthma and allergic diseases. Clin Transl Allergy. 2021;11(7):11. 10.1002/clt2.12061 PMC8420996 34504682

[27] Sousa‐Pinto B , Schünemann HJ , Sá‐Sousa A , et al. Consistent trajectories of rhinitis control and treatment in 16,177 weeks: the MASK ‐air® longitudinal study. Allergy. 2023;78(4):968‐986. 10.1111/all.15574 36325824

[28] Al Rajeh AM , Aldabayan YS , Aldhahir A , et al. Once daily versus overnight and symptom versus physiological monitoring to detect exacerbations of chronic obstructive pulmonary disease: pilot randomized controlled trial. JMIR mHealth uHealth. 2020;8(11):1‐14. 10.2196/17597 PMC7695523 33185560

[29] Mishra T , Wang M , Metwally AA , et al. Pre‐symptomatic detection of COVID‐19 from smartwatch data. Nat Biomed Eng [Internet]. 2020; 4:1208‐1220. 10.1038/s41551-020-00640-6 33208926 PMC9020268

[30] Bousquet J , Anto JM , Sousa‐Pinto B , et al. Digitally‐enabled, patient‐centred care in rhinitis and asthma multimorbidity: the ARIA‐MASK‐air ® approach. Clin Transl Allergy. 2023;13(1):13. 10.1002/clt2.12215 PMC9823305 36705508

[31] Bédard A , Basagaña X , Anto JM , et al. Mobile technology offers novel insights into the control and treatment of allergic rhinitis: the MASK study. J Allergy Clin Immunol. 2019;144(1):135‐143.e6. 10.1016/j.jaci.2019.01.053 30951790

[32] Bédard A , Sofiev M , Arnavielhe S , et al. Interactions between air pollution and pollen season for rhinitis using mobile technology: a MASK‐POLLAR study. J Allergy Clin Immunol Pract [Internet]. 2020;8(3):1063‐1073.e4. 10.1016/j.jaip.2019.11.022 31786252

[33] Stas M , Aerts R , Hendrickx M , et al. Exposure to green space and pollen allergy symptom severity: a case‐crossover study in Belgium. Sci Total Environ [Internet]. 2021; 781: 146682. 10.1016/j.scitotenv.2021.146682 33812114

[34] Kapoor K , Raywood E , Douglas H , et al. Determining resting heart rate in children using wearable activity monitors. Eur Respir J [Internet]. 2019; 54: PA342. http://erj.ersjournals.com/content/54/suppl_63/PA342.abstract

[35] Richman JS , Moorman JR . Physiological time‐series analysis using approximate entropy and sample entropy maturity in premature infants physiological time‐series analysis using approximate entropy and sample entropy. Am J Physiol Hear Circul Physiol. 2000;278(6):H2039‐H2049. 10.1152/ajpheart.2000.278.6.h2039 10843903

[36] Yentes JM , Hunt N , Schmid KK , Kaipust JP , McGrath D , Stergiou N . The appropriate use of approximate entropy and sample entropy with short data sets. Ann Biomed Eng. 2013;41(2):349‐365. 10.1007/s10439-012-0668-3 23064819 PMC6549512

[37] de Vries H , Kamphuis W , Oldenhuis H , van der Schans C , Sanderman R . Moderation of the stressor‐strain process in interns by heart rate variability measured with a wearable and smartphone app: within‐subject design using continuous monitoring. JMIR Cardio. 2021;5(2):e28731. 10.2196/28731 34319877 PMC8524333

[38] Rojo J , Picornell A , Oteros J . AeRobiology: the computational tool for biological data in the air. Methods Ecol Evol. 2019;10(8):1371‐1376. 10.1111/2041-210x.13203

[39] Pfaar O , Bastl K , Berger U , et al. Defining pollen exposure times for clinical trials of allergen immunotherapy for pollen‐induced rhinoconjunctivitis – an EAACI position paper. Allergy Eur J Allergy Clin Immunol. 2017;72(5):713‐722. 10.1111/all.13092 27874202

[40] Warburton DER , Katzmarzyk PT , Rhodes RE , Shephard RJ . Evidence‐informed physical activity guidelines for Canadian adults. Appl Physiol Nutr Metab. 2007;32(S2E):16‐68. 10.1139/h07-123 18213940

[41] Peters A , Perz S , Döring A , Stieber J , Koenig W , Wichmann HE . Increases in heart rate during an air pollution episode. Am J Epidemiol. 1999;150(10):1094‐1098. 10.1093/oxfordjournals.aje.a009934 10568625

[42] Terkelsen AJ , Mølgaard H , Hansen J , Andersen OK , Jensen TS . Acute pain increases heart rate: differential mechanisms during rest and mental stress. Auton Neurosci Basic Clin. 2005;121(1‐2):101‐109. 10.1016/j.autneu.2005.07.001 16081322

[43] Byun S , Kim AY , Jang EH , et al. Detection of major depressive disorder from linear and nonlinear heart rate variability features during mental task protocol. Comput Biol Med [Internet]. 2019; 112: 103381. 10.1016/j.compbiomed.2019.103381 31404718

[44] Lanata A , Valenza G , Nardelli M , Gentili C , Scilingo EP . Complexity index from a personalized wearable monitoring system for assessing remission in mental health. IEEE J Biomed Health Inf US. 2015;19(1):132‐139. 10.1109/jbhi.2014.2360711 25291802

[45] Mather M , Thayer J . How heart rate variability affects emotion regulation brain networks. Curr Opin Behav Sci. 2018;19:98‐104. 10.1016/j.cobeha.2017.12.017 29333483 PMC5761738

[46] Seppänen TM , Alho OP , Seppänen T . Dynamic changes in heart rate variability and nasal airflow resistance during nasal allergen provocation test. J Healthc Eng. 2016;2016:1‐9. 10.1155/2016/1245418 PMC5058568 27196870

[47] Ruiz‐Garcia M , Bartra J , Alvarez O , et al. Cardiovascular changes during peanut‐induced allergic reactions in human subjects. J Allergy Clin Immunol [Internet]. 2021;147:633‐642. 10.1016/j.jaci.2020.06.033 32707226 PMC7858218

[48] Salerno SM , Jackson JL , Berbano EP . Effect of oral pseudoephedrine on blood pressure and heart rate: a meta‐analysis. Arch Intern Med. 2005;165(15):1686‐1694. 10.1001/archinte.165.15.1686 16087815

