# Supplementary material for: Daily allergy burden and heart rate characteristics in adults with allergic rhinitis based on a wearable telemonitoring system
Source: Clin Transl Allergy. 2023 Apr 24;13(4):e12242. doi: 10.1002/clt2.12242 (PMC10126716; doi:10.1002/clt2.12242)
Supplement: Supplementary file 1 — Supporting Information S1 [file CLT2-13-e12242-s001.docx]

Appendix – Daily allergy burden and heart rate characteristics in adults with allergic rhinitis based on a wearable telemonitoring system

# Resting heart rate

**Main analysis (distributed lag models)**

Table E1: Associations between allergy symptom and mood score, and resting heart rate expressed as β coefficient (95% CI) of a linear mixed effects model with random intercepts for participants and including exposure variables at lags of 0, 1 and 2 days together (distributed lag model). Higher symptom score indicates more severe symptoms, higher mood score indicates better mood. Models were adjusted for age, sex, additional allergy to grass and/or dust, time spent in moderate-to-vigorous physical activity, exposure to nitrogen dioxide, exposure to particulate matter with diameter less than 10 μm, medication intake, month, weekend days and pollen seasons of birch, alder and hazel. Also a first-order autocorrelation structure for the residuals was added. Multi-exposure models were additionally adjusted for the other exposure variable at lags of 0, 1 and 2 days.

| Resting heart rate, (beats per minute) | Single-exposure | Multi-exposure |
| --- | --- | --- |
| Allergy symptom score | 0.03 (-0.03 to 0.10) | 0.04 (-0.02 to 0.11) |
| Allergy symptom score (lag 1) | **0.08 (0.02 to 0.15)** | **0.09 (0.02 to 0.16)** |
| Allergy symptom score (lag 2) | -0.06 (-0.12 to 0.00) | -0.06 (-0.13 to 0.01) |
| Mood score | -0.07 (-0.38 to 0.24) | 0.22 (-0.15 to 0.58) |
| Mood score (lag 1) | 0.12 (-0.20 to 0.45) | 0.24 (-0.13 to 0.62) |
| Mood score (lag 2) | 0.13 (-0.19 to 0.44) | 0.03 (-0.35 to 0.40) |

**Sensitivity analysis (single lag models)**

Table E2: Associations between allergy symptom and mood score, and resting heart rate expressed as β coefficient (95% CI) of a linear mixed effects model with random intercepts for participants and including only one lag of the exposure variables at a time (single lag model). Higher symptom score indicates more severe symptoms, higher mood score indicates better mood. Models were adjusted for age, sex, additional allergy to grass and/or dust, time spent in moderate-to-vigorous physical activity, exposure to nitrogen dioxide, exposure to particulate matter with diameter less than 10 μm, medication intake, month, weekend days and pollen seasons of birch, alder and hazel. Also a first-order autocorrelation structure for the residuals was added. Multi-exposure models were additionally adjusted for the other exposure variable at the same lag.

| Resting hear rate, (beats per minute) | Single-exposure | Multi-exposure |
| --- | --- | --- |
| Allergy symptom score | 0.03 (-0.01 to 0.07) | 0.04 (-0.00 to 0.08) |
| Allergy symptom score (lag 1) | **0.07 (0.02 to 0.12)** | **0.08 (0.04 to 0.13)** |
| Allergy symptom score (lag 2) | 0.01 (-0.04 to 0.06) | 0.02 (-0.03 to 0.07) |
| Mood score | 0.03 (-0.20 to 0.26) | 0.22 (-0.05 to 0.49) |
| Mood score (lag 1) | 0.08 (-0.19 to 0.34) | 0.28 (-0.03 to 0.60) |
| Mood score (lag 2) | 0.24 (-0.03 to 0.51) | 0.20 (-0.12 to 0.52) |

# Heart rate sample entropy

**Main analysis (distributed lag models)**

Table E3: Associations between allergy symptom and mood score, and heart rate sample entropy expressed as β coefficient (95% CI) of a linear mixed effects model with random intercepts for participants and including exposure variables at lags of 0, 1 and 2 days together (distributed lag model). Higher symptom score indicates more severe symptoms, higher mood score indicates better mood. Models were adjusted for age, sex, additional allergy to grass and/or dust, time spent in moderate-to-vigorous physical activity, exposure to nitrogen dioxide, exposure to particulate matter with diameter less than 10 μm, medication intake, month, weekend days and pollen seasons of birch, alder and hazel. Also a first-order autocorrelation structure for the residuals was added. Multi-exposure models were additionally adjusted for the other exposure variable at lags of 0, 1 and 2 days.

| Sample entropy, (x10^-2^) | Single-exposure | Multi-exposure |
| --- | --- | --- |
| Allergy symptom score | 0.06 (-0.03 to 0.15) | 0.09 (-0.01 to 0.19) |
| Allergy symptom score (lag 1) | 0.02 (-0.08 to 0.12) | 0.02 (-0.08 to 0.12) |
| Allergy symptom score (lag 2) | -0.06 (-0.15 to 0.04) | -0.04 (-0.14 to 0.06) |
| Mood score | **0.80 (0.34 to 1.26)** | **0.68 (0.13 to 1.23)** |
| Mood score (lag 1) | 0.13 (-0.35 to 0.61) | 0.09 (-0.47 to 0.65) |
| Mood score (lag 2) | 0.35 (-0.12 to 0.82) | 0.43 (-0.13 to 1.00) |

**Sensitivity analysis (single lag models)**

Table E4: Associations between allergy symptom and mood score, and heart rate sample entropy expressed as β coefficient (95% CI) of a linear mixed effects model with random intercepts for participants and including only one lag of the exposure variables at a time (single lag model). Higher symptom score indicates more severe symptoms, higher mood score indicates better mood. Models were adjusted for age, sex, additional allergy to grass and/or dust, time spent in moderate-to-vigorous physical activity, exposure to nitrogen dioxide, exposure to particulate matter with diameter less than 10 μm, medication intake, month, weekend days and pollen seasons of birch, alder and hazel. Also a first-order autocorrelation structure for the residuals was added. Multi-exposure models were additionally adjusted for the other exposure variable at the same lag.

| Sample entropy, (x10^-2^) | Single-exposure | Multi-exposure |
| --- | --- | --- |
| Allergy symptom score | 0.04 (-0.02 to 0.10) | **0.08 (0.01 to 0.14)** |
| Allergy symptom score (lag 1) | 0.01 (-0.06 to 0.09) | 0.02 (-0.05 to 0.10) |
| Allergy symptom score (lag 2) | -0.02 (-0.10 to 0.05) | -0.00 (-0.08 to 0.07) |
| Mood score | **0.79 (0.45 to 1.14)** | **0.79 (0.38 to 1.20)** |
| Mood score (lag 1) | 0.30 (-0.10 to 0.71) | 0.23 (-0.24 to 0.71) |
| Mood score (lag 2) | **0.52 (0.11 to 0.93)** | 0.47 (-0.01 to 0.95) |
